# Anxiety, depression symptoms, and psychological resilience among hospitalized COVID‐19 patients in isolation: A study from Wuhan, China

**DOI:** 10.1002/brb3.3274

**Published:** 2023-11-01

**Authors:** Li Chen, Zhenmeng Wang, Dan Liu, Dandan He, Shulin Du, Zezhi Li, Shuyun Li, Yuehong Sheng

**Affiliations:** ^1^ Third Affiliated Hospital Naval Medical University Shanghai China; ^2^ Department of Psychiatry The Affiliated Brain Hospital of Guangzhou Medical University Guangzhou China; ^3^ Guangdong Engineering Technology Research Center for Translational Medicine of Mental Disorders Guangzhou China; ^4^ Department of Emergency Medicine The Affiliated Brain Hospital of Guangzhou Medical University Guangzhou China

**Keywords:** anxiety, COVID‐19, depression, psychological resilience

## Abstract

**Objective:**

To investigate the status of anxiety, depression, and psychological resilience among individuals with COVID‐19, and their interrelationships to provide a scientific basis for developing psychological intervention strategies for these patients.

**Methods:**

A total of 126 patients with COVID‐19 who were admitted to Wuhan Huoshenshan Hospital were recruited in this study. A comprehensive survey was conducted using a general information questionnaire, the Self‐Rating Anxiety Scale, the self‐rating depression scale, and the Chinese version of the psychological Connor–Davidson resilience scale; a questionnaire‐based survey was conducted.

**Results:**

Significant differences in anxiety scores were observed among COVID‐19 patients with different education levels and the number of immediate family members. The differences in depression scores were noted among patients of different age groups, and marital statuses were also significant. The total psychological resilience score and the scores of all dimensions are negatively correlated with anxiety and depression. Furthermore, the patient's gender, the number of immediate family members, and the psychological resilience dimensions are associated with the severity anxiety of patients. Patient age and psychological resilience are associated with the depression level of patients.

**Conclusion:**

Patients with COVID‐19 exhibit elevated levels of both anxiety and depression. Notably, psychological resilience emerges as a protective factor against the development of anxiety and depression.

## Abstract 2

COVID‐19 patients have higher levels of anxiety and depression symptoms. Life conditions of COVID‐19 patients are related to anxiety and depression symptoms. Psychological resilience prevent the anxiety and depression symptoms.

## 1 INTRODUCTION

The global dissemination of the coronavirus (COVID‐19) is unprecedented, causing high morbidity and mortality worldwide (Li et al., 2020). Along with the development of the disease, healthcare professionals have accumulated a great amount of experience and knowledge of the disease, which has helped establish medical systems to better respond to the pandemic. COVID‐19 exhibits a wide spectrum of manifestations, ranging from asymptomatic infection to life‐threatening multiorgan disease, exerting a profound impact on the mental health of the population, including the patients infected with COVID‐19, healthcare personnel, and even individuals who remain unaffected by the virus (Huang et al., 2021; Sheng et al., 2022; Wang et al., 2021; Zhou et al., 2020).

Certainly, the psychological stress induced by the COVID‐19 pandemic has exceeded that in the previous coronavirus outbreaks (Deng et al., 2020). For that reason, it is possible that COVID‐19 patients who require intensive care could potentially become a high‐risk population for developing psychiatric disorders (Sommer & Bakker, 2020). Furthermore, previous evidence has shown that hospitalized patients were more likely to have persistent symptoms after COVID‐19 than asymptomatic or home‐treated subjects (Craparo et al., 2022).

COVID‐19 has a negative impact on the individuals’ quality of life children and psychiatric admissions to emergency department (Ambrosetti et al., 2021; Nobari et al., 2021). Especially, COVID‐19 lockdown can impact individuals’ mental health (Amerio et al., 2021). Psychological responses to infectious diseases include maladaptive behavior, emotional distress, and defensive reactions (Taylor, 2019). Few studies have reported that COVID‐19 patients show severe symptoms of anxiety, depression, and post‐traumatic and somatic symptoms (Romero‐Sánchez et al., 2020). For example, Janiri et al. (2021) revealed that 30.2% of patients afflicted with COVID‐19 had post‐traumatic stress disorder (PTSD). Lee et al. (2021) showed that the COVID‐19 patients displayed a 20.71‐fold risk of anxiety, 14.45‐fold risk of depression, 5.65‐fold risk of somatic symptoms, and 24.16‐fold risk of PTSD. However, these studies primarily relied on telephonic interviews or online questionnaires, leaving a gap in our understanding of mental health challenges experienced by infected patients during the hospitalization in the first wave of COVID‐19 in Wuhan city. Furthermore, Craparo et al. (2022) demonstrated that high levels of anxiety and depression predicted the PTSD symptoms in individuals who have recovered from COVID‐19, and they found that individuals hospitalized by COVID‐19 are more at risk of developing intrusion and hyperarousal symptoms than those who never needed hospital care. Therefore, it is important to investigate the anxiety and depression symptoms of patients hospitalized by COVID‐19.

To ensure that all suspected and confirmed cases are admitted and treated in an isolated hospital, the new hospital named “Huoshenshan” was designed to treat patients suffering from COVID‐19 (China.org.cn, 2020), and the patients who were admitted to the Huoshenshan Hospital were all COVID‐19 confirmed cases. During the first wave of COVID‐19, numerous patients exhibited fear and a range of other negative emotions. They were susceptible to feelings of panic, anxiety, and depression. In some severe cases, the COVID‐19 patients further developed PTSD. Psychological resilience, proposed by American psychologist Anthony in the 1970s, is referred to the ability of an individual to actively adapt and effectively cope with adversity, trauma, pain, or tragedy (Jackson et al., 2007). Good mental flexibility is critical for individuals to maintain a healthy and stable mental status. For the population of COVID‐19 patients, the relationship between psychological flexibility and negative emotions is still unclear. How to strengthen the exploration of psychological resilience during the psychological intervention of patients and promote their effective response and adaptation is a new issue facing our nursing workers.

We hypothesized that psychological resilience is correlated with anxiety and depression of confirmed COVID‐19 patients. This study presented the basic data on mental health concerns for hospitalized patients with new coronary pneumonia during the first wave of COVID‐19 in Wuhan, by investigating the current status and correlation of anxiety, depression, and psychological resilience levels of confirmed cases in Wuhan Huoshenshan Hospital. It may provide guidance on psychological interventions for major public health incidents in the future.

## 2 SUBJECTS AND METHODS

### 2.1 Subjects

A total of 130 patients diagnosed with COVID‐19 were recruited from Huoshenshan Hospital from February 10, 2020 to April 3, 2020, using computerized random number generation. The inclusion criteria were as follows: (1) met the diagnostic criteria for COVID‐19; (2) have the ability to take care of themselves; (3) age ≥18 years old. Exclusion criteria were as follows: (1) with a history of neurological or mental illness; (2) with severe other physical diseases; (3) progression or deterioration of the COVID‐19 infection; (4) received antidepressant or antianxiety agents within 1 month.

During the course of the study, four patients were transferred to the intensive care unit due to worsening conditions. Eventually, a total of 126 patients (70 males and 56 females) were included in the study with a mean age of 56.05 ± 12.68 and an illness duration of 3.49 ± 0.90 days. Among them, 114 patients were city residents, whereas 12 patients were rural residents.

### 2.2 Clinical interview

Two nurses in the quarantine area interviewed the patients and interpreted the questionaries for them. After completing the interview and assessments, photos taken by the nurses were uploaded to the computer for data storage in the non‐contaminated area. For patients who had difficulties in filling out the questionaries, the research team explained the questionaries to them following the standard requirements without giving any hints and assisted them in filling out the questionaries based on their own judgment. The questionnaires were collected and checked in the hospital.

### 2.3 Instruments for assessments

The self‐designed questionnaire included information on age, gender, location of long‐term residence, marital status, education level, monthly income, illness duration, and number of infected family members, which may affect anxiety or depressive symptoms.

Connor–Davidson Resilience Scale (CD‐RISC) was applied to assess the psychological resilience of the patients. The Cronbach *α* coefficient of the scale is .89, and the test–retest reliability is 0.87 (Connor & Davidson, 2003). This study used the Chinese version of the psychological resilience scale (Yu & Zhang, 2007), containing 25 items of the original scale and consisting of 3 dimensions: tenacity, strength, and optimism. Each item was rated on a 5‐point scale (0–4), which consisted of a 5‐point range of responses as follows: not true at all (0), rarely true (1), sometimes true (2), often true (3), and true nearly all of the time (4). The scale was rated based on how the participants have felt over the past month. The total score ranges from 0 to 100, with higher scores reflecting greater resilience.

The Self‐Rating Anxiety Scale (SAS) was applied to assess the severity of anxiety of the participants, containing 20 items, including 15 forward scoring items and 5 reverse scoring items. The full score is 100 points. The higher score suggests the more severe anxiety symptoms. The upper limit of the SAS score for a normal condition is 50 points. A total of 50–59 points indicate mild anxiety, 60–69 points indicate moderate anxiety, and ≥70 points indicate severe anxiety.

Self‐rating depression scale (SDS) was applied to assess the severity of depressive symptoms of participants, containing 20 items, including 10 forward scoring items and 10 reverse scoring items. The full score is 100 points. A higher score suggests more severe depressive symptoms. The upper limit score of SDS for a normal condition is 53 points. A total of 53–62 points indicate mild depression, 63–72 points indicate moderate depression, and ≥73 points indicate severe depression.

### 2.4 Statistical analysis

SPSS21.0 data analysis software was used for statistical analysis, including descriptive statistical analysis, one‐way analysis of variance, the Pearson correlation analysis, and multiple stepwise regression analysis. *p* < .05 indicates that the difference is statistically significant. The sample sizes were determined by using GPower 3.1, and the statistical power was more than .8.

## 3 RESULTS

### 3.1 Anxiety, depression, and psychological resilience in the hospitalized COVID‐19 patients

The anxiety score of the patients was 51.27 ± 7.67, including 65 cases (51.6%) of mild anxiety, 11 cases (8.7%) of moderate anxiety, and 3 cases (2.4%) of severe anxiety. The depressive symptom score of the patients was 52.68 ± 9.91, including 55 cases of mild depression (43.7%), 19 cases of moderate depression (15.1%), and 2 cases of severe depression (1.6%). The CD‐RISC score of the patients was 68.36 ± 18.44. The score of three dimensions, including tenacity, strength, and optimism, was 35.19 ± 10.12, 22.90 ± 6.15, and 10.26 ± 3.30, respectively.

### 3.2 Univariate analysis of anxiety and depression in the COVID‐19 patients

As shown in Table 1, there was a significant difference in anxiety scores among some different clinical characteristics. Women have higher anxiety scores than men (*p* = .008). Patients in a rural area had higher anxiety scores than patients in an urban area (*p* = .048). Patients with lower education were more likely to experience anxiety (*p* = .023). More infected family members were more likely to have anxiety symptoms (*p* = .033). For depressive symptoms, there was a significant difference in depression scores in ages (*p* = .008) and marriage status (*p* = .003).

**TABLE 1:** Univariate analysis of anxiety, depressive symptoms in COVID‐19 patients (*n* = 126).

	*n*	Anxiety symptoms	*t*/*F*	*p*	Depressive symptoms	*t*/*F*	*p*
Age			1.86	.16		5.00	**.008**
≤44 years	24	48.75 ± 7.15			47.29 ± 10.45		
45–59 years	51	52.38 ± 8.28			53.16 ± 10.16		
≥60 years	51	51.35 ± 7.10			54.73 ± 8.59		
Sex			−2.70	**.008**		−.09	.93
Male	70	49.66 ± 6.71			52.61 ± 9.95		
Female	56	53.28 ± 8.34			52.77 ± 9.96		
Residence			−1.99	**.048**		−1.08	.28
City	114	50.83 ± 7.65			52.37 ± 10.04		
Rural area	12	55.42 ± 6.79			55.63 ± 8.47		
Marital status			2.56	.08		5.95	**.003**
Single	6	45.63 ± 9.84			39.58 ± 13.43		
Married	111	51.80 ± 7.61			53.29 ± 9.54		
Divorced/Widows	9	48.47 ± 4.83			53.89 ± 5.94		
Education			3.91	**.02**		2.18	.12
Primary and secondary school	55	53.34 ± 7.66			54.61 ± 8.43		
Senior school or technical secondary school	37	50.17 ± 8.06			52.03 ± 10.16		
College and above	34	49.12 ± 6.50			50.26 ± 11.43		
Income			1.08	.36		.99	.40
˂2000	22	53.07 ± 9.35			51.65 ± 11.14		
2000–3999	53	51.75 ± 8.19			53.58 ± 9.46		
4000–4999	15	51.08 ± 5.53			55.33 ± 8.30		
>5000	36	49.55 ± 6.33			50.87 ± 10.38		
Sickness of family members			1.28	.202		−.30	.77
None	61	52.12 ± 7.33			52.42 ± 9.63		
Yes	65	50.37 ± 7.97			52.95 ± 10.29		
Numbers of sickness of family members			3.00	**.03**		2.27	.08
0	59	50.15 ± 7.94			52.82 ± 10.43		
1	39	50.87 ± 6.42			50.99 ± 10.27		
2	17	52.13 ± 7.93			51.62 ± 8.17		
>3	11	57.39 ± 7.78			59.55 ± 4.98		

### 3.3 The association of psychological resilience with anxiety and depression in COVID‐19 patients

As shown in Table 2, CD‐RISC total score was negatively correlated with anxiety (*r* = −.37, *p* < .001) and depression (*r* = −.30, *p* = .001), even after the Bonferroni correction. It suggested that the lower the psychological resilience level of COVID‐19 patients, the more severe anxiety and depression they have.

**TABLE 2:** Association of psychological resilience with anxiety and depression in patients with COVID‐19.

	Anxiety symptoms		Depressive symptoms	
	*r*	*p*	*r*	*p*
Tenacity	−.32	<.001	−.27	.002
Strength	−.43	<.001	−.33	<.001
Optimism	−.26	.004	−.27	.003
Total score	−.37	<.001	−.30	.001

Regarding the three dimensions of CD‐RISC, we found that tenacity, strength, and optimism were all negatively associated with anxiety and depression (both *p* < .05), even after the Bonferroni correction.

The psychological resilience dimension can predict the anxiety of patients with COVID‐19 (*p* < .001), and the patient's age and psychological resilience dimension can predict their depression levels (*p* < .001). The details are shown in Table 3.

**TABLE 3:** The association of psychological resilience with anxiety and depressive symptoms.

	*β*	*t*	*p*	*Β* (CI 95%)
Anxiety symptoms				
Sex	3.47	2.78	.006	2.35–4.59
Numbers of sickness of family members	1.72	2.67	.009	.41–3.03
Strength	−.41	−4.02	<.001	−.49 to −.33
Depressive symptoms				
Age	2.36	2.18	.03	.68–3.04
Strength	−.65	−4.93	<.001	−.72 to −.58

*Note*: Anxiety regression model: *R* ^2^ = 0.21, adjusted *R* ^2^ = 0.17; depression regression model: *R* ^2^ = 0.22, adjusted *R* ^2^ = 0.21.

## 4 DISCUSSION

This study had several main findings: (1) Psychological resilience is negatively correlated with anxiety and depression; (2) the gender, the number of immediate family members, and the psychological resilience dimensions are associated with the severity anxiety of patients, whereas the age and psychological resilience are associated with the depression level of patients.

Patients hospitalized for COVID‐19 treatment encounter a range of mental health challenges arising from factors, such as isolation, feelings of loneliness, heightened anxiety, depression, phobias, concerns about the progression of the disease, and insufficient resources and information upon admission (Brooks et al., 2020). During hospitalization, patients continue to experience additional stresses related to COVID‐19, which may create new psychological problems. It has been reported that the prevalence of anxiety and depression in COVID‐19 inpatients was 16.3%–55.3% and 22.9%–60.2%, respectively (Kim et al., 2021; Ma et al., 2020; Paz et al., 2020; Zhang et al., 2020). However, these studies were based on telephonic interviews or online questionnaires. Our study was conducted through face‐to‐face interviews with hospitalized infected patients during the first wave of COVID‐19 in Wuhan, China.

### 4.1 Anxiety and depression in patients with COVID‐19

The incidence rates of anxiety and depression were 62.7% and 60.4%, respectively. Confronting COVID‐19 constitutes a potent psychological stressor for patients. This stress is fueled by an array of factors, including enforced isolation, instances of familial cluster infections, apprehensions concerning the well‐being of other family members, concerns about potential complications in prognosis, and the mortality rate linked to the disease. These cumulative stressors can trigger psychological strain among patients, manifesting as varying degrees of anxiety and depression.

This study revealed notable variations in anxiety scores among COVID‐19 patients based on factors, such as gender, place of residence, educational attainment, and the number of immediate family members who are also patients. Likewise, differences in depression scores emerged among patients based on age and marital status. The levels of anxiety and depression demonstrated diversity among patients with distinct sociodemographic attributes.

Specifically, female patients exhibited higher levels of anxiety and depression compared to their male counterparts. This discrepancy may stem from inherent psychological traits that render females more susceptible to negative emotions during sudden, major epidemics. Additionally, females might experience challenges in cognitive acceptance of the epidemic's reality. Furthermore, individuals residing in rural areas with a monthly income below 2000 units exhibited significantly elevated anxiety levels. This could be attributed to income reduction triggered by disruptions in agricultural product transportation and sales due to the epidemic's impact. These findings underscore the significance of considering sociodemographic characteristics when assessing and addressing the psychological impact of major outbreaks like COVID‐19.

Furthermore, it is worth noting that rural residents often grapple with dissatisfaction stemming from inadequate work conditions, lower wages, limited resources, and suboptimal environments, which can contribute to poorer mental health outcomes. Different educational levels among patients yield varying degrees of anxiety and depression. This discrepancy might be linked to well‐educated patients’ propensity for seeking a comprehensive understanding of the disease, their openness to new information and knowledge, and their ability to adapt to novel circumstances. These factors enable them to engage in effective communication with medical staff, ultimately aiding in stress alleviation.

Conversely, patients with lower levels of education might encounter difficulties acquiring new information and establishing effective communication. Their lack of a nuanced comprehension of the epidemic can lead to feelings of helplessness. The occurrence of family clusters during the epidemic has notably elevated anxiety and depression levels, particularly among individuals with three or more immediate family members diagnosed with COVID‐19. The altered dynamics of family interaction due to members being isolated in different hospitals intensify concerns and feelings of powerlessness, exacerbating mental strain for patients.

Hence, it is imperative for clinical nursing practice to prioritize emotional communication with COVID‐19 patients, particularly focusing on female patients, those residing in rural areas with limited education or income, and those who have several immediate family members diagnosed with COVID‐19. Tailored nursing interventions that adjust the communication approach between nurses and patients should be considered to better address these specific challenges. For example, CICARE (Connect, Introduce, Communicate, Ask, Respond, Exit) communication mode can be used to establish a good relationship between nurses and patients and to formulate a specific communication plan, which will benefit from distributing the knowledge about the etiology, transmission route, nursing process, disinfection, and isolation during the communication. Besides, giving some examples of the cured cases can eliminate the patient's fear.

A regular daily schedule can be made, and at the same time, aerobic exercises such as Tai Chi and Baduanjin can be organized. Those exercises have low requirements for venues and facilities and are easy to learn. Studies have shown that Taijiquan and Baduanjin can effectively improve anxiety and depression in patients with coronary heart disease (Solianik et al., 2021; Zhang et al., 2021). To the isolated patients with mild type or common types of the disease, life intervention can help adjust their mentality, reduce their anxiety and depression, improve their quality of life, and facilitate disease recovery (Zhang et al., 2021). Assisting the patient in communicating closely with the most trusted and close family members can reduce the patient's negative psychological emotions and help the patient restore the family support system.

### 4.2 Analysis of the psychological resilience in COVID‐19 patients

The resilience score of this group of COVID‐19 patients was lower than the score of the general population in the United States (Dubey et al., 2015), and general adults in China (Yu et al., 2009), suggesting that the mental resilience of the COVID‐19 patients is low in this study. Likely because of the social panic since the outbreak of COVID‐19 in December 2019 due to its strong contagion, rapid development, a certain fatality rate, and the lack of effective treatment methods. Diagnosed patients are treated in isolated hospitals with limited activities, and no visiting or accompanying is allowed. They almost lose connection with the outside and are prone to be psychologically stressed. In addition, the patients in this study are all patients diagnosed with COVID‐19 for the first time, and they have not fully adapted to the changes of life brought by the disease. Under the various types of stress, the patients generally have anxiety, depression, and negative emotions, which in turn affect their psychological resilience. Studies have shown that resilience plays an important role as a protective factor for mental health during the COVID‐19 outbreak (Sugawara et al., 2022). The research of Joyce et al. (2010) believes that individuals with a higher level of psychological resilience are able to respond in a more effective way. These all suggest that in our clinical practice, medical staff should be aware of the significance of psychological resilience to patients’ physical and mental health and pay close attention to the level of psychological resilience of the patients. As frontline workers, we must assess the level of psychological resilience of patients timely and dynamically, communicate with patients proactively and effectively, and provide individualized and targeted nursing interventions. Doctor and nurse joint rounds should be established. It is important to help patients to build confidence and hope for actively cooperating with treatment, by sharing the updated and effective treatment plans as well as the information of recovered and discharged patients, but not the negative and wrong information on the public media. Every progress of patients made should be encouraged, which can help to restore and improve the psychological resilience level of the patients, and to promote their positive coping ability.

### 4.3 Analysis of influencing factors of anxiety and depression in COVID‐19 patients

This study shows that the total score and each dimension score of COVID‐19 patients are negatively correlated with anxiety and depression, indicating that the higher the level of psychological resilience of patients, the lower the degree of anxiety and depression, and vice versa. The tenacity dimension negatively predicts anxiety and depression, suggesting that tenacity is a protective factor for mental health, and psychological resilience is an ability to cope with adversity and pressure. The higher the ability of the tenacity, the more determined and persistent when facing adversity or pressure. The COVID‐19 patients with higher tenacity can treat the disease with a positive attitude, adjust and respond in an effective way with positive emotions so that the spirit and psychology are in a healthy state, which reduces the negative impact induced by the stress and benefits from adjusting the negative emotions such as anxiety and depression, thereby reducing the occurrence of anxiety and depression. On the contrary, the patients with lower tenacity ability treat the disease with a negative attitude and cannot effectively adjust and respond, which is harmful to reducing the impact of the stress, and facilitates the occurrence of anxiety and depression. Strength and optimism were involved in the regression equation, likely because the two factors have a certain relationship with tenacity, and there is an interactive effect.

There were several limitations that should be concerned. First, this was a cross‐sectional study and causality could not be established. Further prospective follow‐up studies are needed to clarify causality. Second, the patients in this study were assessed for depression and anxiety symptoms using self‐rating scales and lacked scales such as HAMD17 and HAMA. Third, the severity of respiratory symptoms of COVID‐19 patients was not assessed and may also affect the patient's anxiety, depressive symptoms, and psychological resilience.

Taking together, it becomes imperative for frontline nursing personnel to prioritize psychological resilience as a focal point during their clinical practice. When engaging in psychological interventions for patients, a heightened emphasis on the protective influence of psychological resilience is warranted. By enhancing psychological resilience, individuals grappling with anxiety and depression can experience a reduction in their psychological distress. This improvement enables them to effectively respond to and adapt to the challenges posed by COVID‐19, ultimately fostering mental well‐being and facilitating their journey toward recovery from the illness.

## AUTHOR CONTRIBUTIONS

Li Chen and Zhenmeng Wang contributed to data analysis, interpretation, and manuscript writing. Li Chen and Yuehong Sheng were responsible for the experimental design and carrying out the experiments. Dan Liu, Dandan He, and Shulin Du performed data acquisition. Yuehong Sheng, Shuyun Li, and Zezhi Li conceived the study and supervised. All authors contributed to the article and approved the submitted version.

## CONFLICT OF INTEREST STATEMENT

The authors declared no potential conflicts of interest.

## FUNDING INFORMATION

The Traditional Chinese Medicine Bureau of Guangdong Province, Grant/Award Number: 20222178; Science and Technology Commission of Shanghai Municipality, Grant/Award Number: 20XD1425300; National Natural Science Foundation of China, Grant/Award Number: 81100312

### PEER REVIEW

The peer review history for this article is available at https://publons.com/publon/10.1002/brb3.3274.

## Data Availability

Data will be made available on request.
